# Obturator Nerve Schwannoma as a Mimic of Ovarian Malignancy

**DOI:** 10.1155/2017/9724827

**Published:** 2017-12-06

**Authors:** Tyler Gleason, Brian H. Le, Kirthik Parthasarathy, Bernice Robinson-Bennett

**Affiliations:** ^1^Department of Medicine, Reading Health System, West Reading, PA 19611, USA; ^2^Department of Pathology, Reading Health System, West Reading, PA 16911, USA; ^3^Department of Obstetrics & Gynecology, Gynecologic Oncology, Reading Health System, West Reading, PA 19611, USA

## Abstract

The obturator nerve is an extremely rare location for schwannomas to originate, and such diagnosis is typically not considered among the imaging diagnostic possibilities for a cystic-solid pelvic mass. A 63-year-old female with a known pelvic mass presented with increasing pelvic pain. The mass, which had been followed by serial imaging over five years, was described showing mixed solid and cystic components, likely arising from the left ovary. Although the key diagnosis to be excluded was a primary ovarian malignancy, the patient chose to pursue active surveillance. Over the five years of close observation, the lesion increased slowly, while her CA-125 level showed no significant elevation. Increase in size of the mass and worsening pain and concern for a gynecologic malignancy on MRI led her to ultimately consent to a hysterectomy with bilateral salpingooophorectomy. During the surgery, the mass was noted to be contiguous with the left obturator nerve. Pathologic evaluation revealed a schwannoma (WHO grade I). The patient's postsurgical course was uneventful, without residual weakness in the left adductor muscles.

## 1. Introduction

The differential diagnosis for an unsampled, chronic pelvic mass is broad and includes various gynecologic causes such as benign adnexal cyst, endometriosis, and primary ovarian neoplasms including epithelial, sex chord-stromal, and germ cell tumors. Nongynecologic causes include colorectal masses, lymphoproliferative processes, and soft-tissue tumors [1]. Among the various etiologies, nerve sheath tumors are exceedingly rare and include schwannoma, neurofibroma, perineurioma, and malignant peripheral nerve sheath tumor [2]. Consideration for this latter scenario is especially nuanced by the fact that preoperative imaging may not clearly identify that the origin of a lesion is question as arising from a nerve, such as the obturator nerve; as such, nerve sheath tumors are typically not entertained in the differential diagnosis, giving rise to elevated concerns for primary ovarian malignancy.

## 2. Case Presentation

A 63-year-old female with a history of coronary artery disease, diabetes mellitus type 2, and a slowly but progressively enlarging pelvic mass presented with a chief complaint of worsening pelvic pain. She had been clinically and radiographically followed for the pelvic mass over the past five years but had previously opted for observation instead of surgical intervention.

The mass was first noted on a transvaginal ultrasound performed five years priorly. Ultrasound at that time demonstrated a soft-tissue nodule with a cystic component adjacent to the left lower uterine segment, measuring 2.7 × 2.1 × 2.9 cm that was not present on a prior CT scan of the pelvis from the past ten years. To further explore the mass, magnetic resonance imaging (MRI) of the pelvis was performed, which demonstrated a 2.6 × 2.1 × 2.7 cm mass adjacent to the left pelvic sidewall at the level of the left acetabulum. The mass demonstrated hypointensity on T1-weighted imaging, hyperintensity on T2-weighted imaging, and avid peripheral wall enhancement with gadolinium contrast administration.

**Figure 1:**
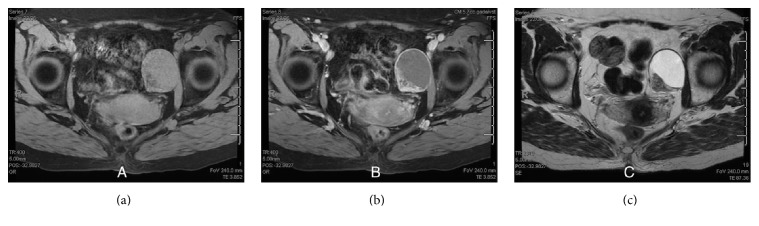
MRI of the pelvic mass with (a) T1, (b) T1 + contrast, and (c) T2 images.

**Figure 2:**
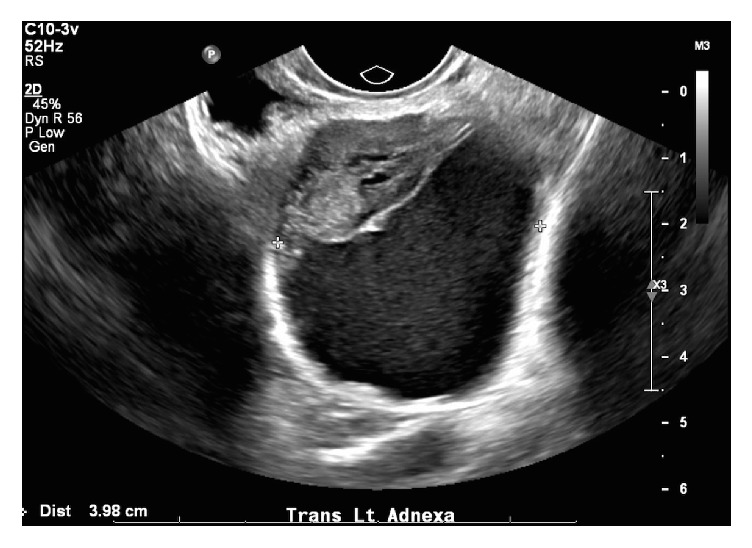
Ultrasound of the left adnexal mass in transview.

Due to patient preference, the mass was followed with serial pelvic exams, imaging, and CA-125 levels. Four CA-125 levels were checked over the next four years and ranged within 3.7–5.1 Units/millilitre (U/mL) (Reference Range: 0.0–35.0 U/mL). Repeat MRI from this most recent encounter showed an interval increase in the size, which now measured 4.3 × 3.8 × 4.1 cm (Figure 1). The mass was heterogeneously isointense and hypointense on T1-weighted imaging and hyperintense on T2-weighted sequence with fat suppression and showed avid rim enhancement on the contrast-enhanced T1-weighted sequence. As her pain continued to worsen, the patient consented to surgical intervention. Repeat ultrasound prior to surgery revealed a 5.5 × 4.6 × 4.0 cm mixed cystic and solid left adnexal mass (Figure 2).

To address her pelvic pain, the patient underwent exploratory laparotomy with hysterectomy and bilateral salpingooophorectomy, with the expectation that the mass in question would be revealed to reflect a primary left ovarian malignancy. On intraoperative exploration, the mass was noted to be contiguous with the left obturator nerve, either invading into the nerve or arising from the nerve itself. The uterus, fallopian tubes, and ovaries were removed to facilitate ease of resection of the retroperitoneal mass. The pelvic mass was separated from the left obturator nerve, which was left intact. Intraoperative consultation, performed on the 4.5 × 2.5 × 1.5 cm mass, showed a spindle-cell tumor.

**Figure 3:**
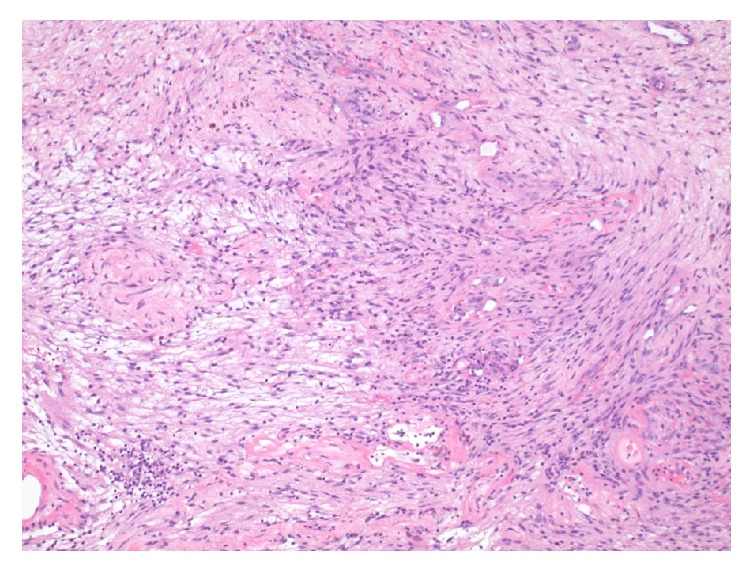
Histologic section showing a bland-appearing, spindled cell proliferation with regions of higher cellularity (Antoni A) interlaced with those of lesser cellularity (Antoni B) (H&E stain, 200x original magnification).

**Figure 4:**
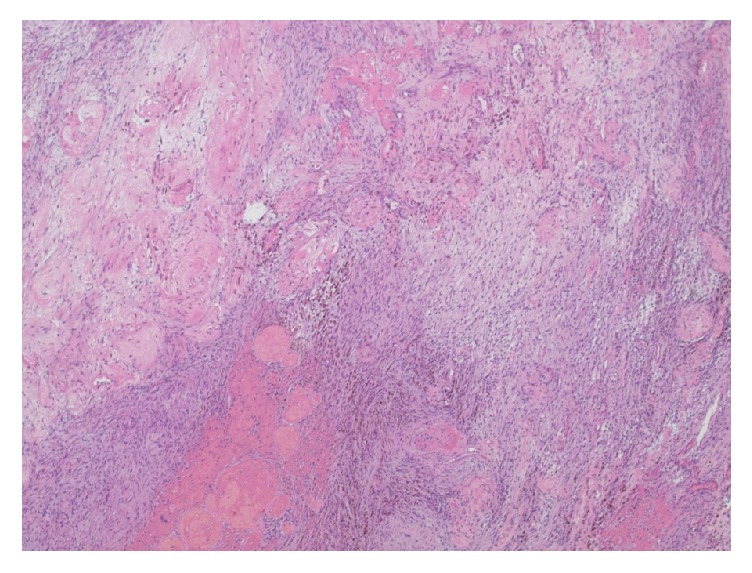
Histologic section showing prominent, hyalinized blood vessels with admixed hemosiderin (“ancient changes”) (H&E stain, 100x original magnification).

**Figure 5:**
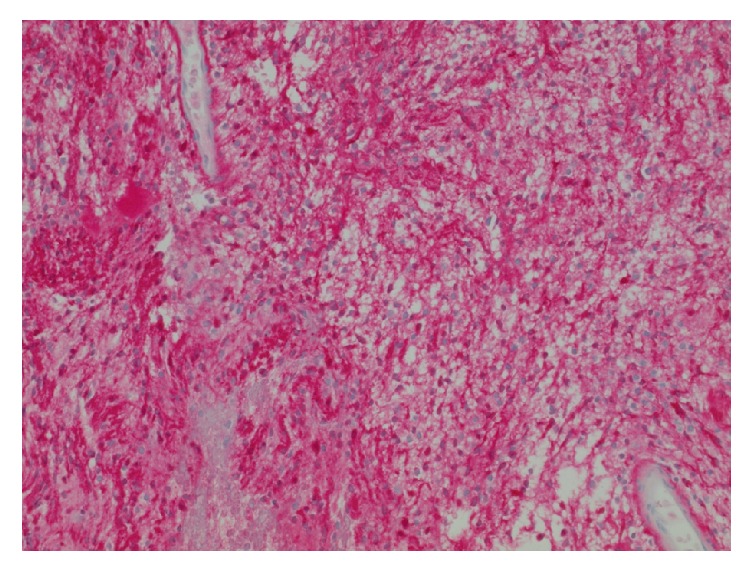
Diffuse immunoreactivity for S-100 protein in neoplastic cells confirms Schwann cell origin of the tumor (200x original magnification).

Permanent histologic sections of the mass demonstrated a proliferation of spindled cells with a fascicular architectural configuration. There are regions of higher, more compact cellularity interlaced with areas that are more loosely cellular, corresponding to the Antoni A and Antoni B patterns (Figure 3). There are thickened, hyalinized blood vessels and patchy foci of hemosiderin deposition (Figure 4). Within the lesion, there are no regions of increased mitotic activity or necrosis. Neoplastic cells show diffuse reactivity (nuclear and cytoplasmic) for the S-100 protein (Figure 5). Markers generally associated with muscle differentiation (desmin, myosin smooth muscle) are negative, as are those associated with epithelial and perineurial differentiation (cytokeratin, epithelial membrane antigen). The global morphologic features, along with immunophenotype, are diagnostic of schwannoma of WHO grade I.

The patient tolerated the procedure well and was discharged to home on postoperative day three. She demonstrated no deficits in the muscles of adduction in the left lower extremity. Her postoperative follow-up at two months revealed neurologic deficits, with resolution of her pelvic pain.

## 3. Discussion

A diagnosis of a obturator nerve sheath tumor before resection is extremely rare, with only one reported case in the literature to date [3]. It is much more common for these tumors to masquerade as another lesion, with definitive diagnosis rendered only after histopathologic examination. Nevertheless, there are certain findings on MRI that may point to a nerve sheath tumor. In particular, on MRI, schwannomas are round with sharply demarcated borders; they appear hypointense or isointense on T1-weighted imaging and hyperintense on T2-weighted imaging; some tumors demonstrate avid rim enhancement while others demonstrate a target-like pattern with greater central than peripheral enhancement [4].

Obturator nerve schwannomas are exceedingly rare, with only a handful cases published worldwide in the English language, with the first published in 1998 [5]. Subsequent individual case reports and one series of 6 cases have commented on experiences with laparascopic and robotic resections of obturator nerve schwannomas [3, 6, 7]. Of note, this is the first case to our knowledge to discuss the benefits of preoperative contrast-enhanced MRI and postoperative pathologic analysis of these exceedingly rare tumors.

In the preoperative imaging of unknown pelvic masses, ultrasound remains the initial imaging modality of choice, as it can be performed more quickly and is more affordable than MRI, while offering similar diagnostic sensitivity [8], where MRI offers a comparative advantage to ultrasound is that it provides much greater specificity. One prospective study found that the sensitivity for ultrasound and MRI in detecting ovarian malignancy is similar at 100% versus 96.6%, respectively, but that MRI has much more specificity than ultrasound at 83.7% versus 39.5%, respectively [9]. Therefore, MRI is most useful in women with low risk of malignancy with indeterminate lesions on ultrasound.

In correlation with the imaging characteristics, schwannomas are typically encapsulated nerve sheath tumors composed entirely of well-differentiated Schwann cells. They usually grown in an extraneural configuration and, as such, may be amenable to resection without compromising the underlying nerve. In contrast, other peripheral nerve sheath tumors, such as neurofibromas, tend to grow in an infiltrative, intraneural fashion, increasing the risk neural compromise with attempts at complete resection. As neurofibromas show a mixture of Schwann cells, fibroblasts, and pericytes, immunohistochemistry for S-100 protein would demonstrate only patchy reactivity, as opposed to the diffuse reactivity that is expected for schwannomas. This pattern of immunoreactivity, along with morphologic characteristics, further facilitates the histopathologic distinction between these two entities [10, 11].

In summary, this rare case of obturator nerve schwannoma illustrates a few salient points to consider when evaluating and managing a pelvic mass lesion:

- Although obturator nerve sheath tumors are rare, they should be considered in the radiographic differential diagnosis of a pelvic tumor.
- Contrast-enhanced MRI can be a useful imaging modality in differentiating nerve sheath tumors from other pelvic tumors, particularly when used in conjunction with ultrasound.
- It is important to pathologically distinguish schwannomas from neurofibromas; whereas schwannomas are usually extraneural and may be amenable to gross total resection without neurologic compromise, neurofibromas are typically intraneural and attempts at total resection may give rise to neurologic deficits.
- Experience with the few obturator nerve schwannomas that have been characterized have mostly described a benign behavior, although transformation to a malignant peripheral nerve sheath tumor can occur.
- Obturator nerve schwannomas, when occurring in isolation, are typically sporadic tumors that do not reflect a genetic predisposition syndrome such as neurofibromatosis.
